# Supplementary material for: Causal interplay between lactose intolerance and gut microbiota: a combined bidirectional Mendelian randomization and in vivo validation study
Source: Front Nutr. 2026 Jun 1;13:1803337. doi: 10.3389/fnut.2026.1803337 (PMC13265576; doi:10.3389/fnut.2026.1803337)
Supplement: Supplementary file 1 [file Data_Sheet_1.zip › supplementary materials/Forward/leave one out cross validation/ebi-a-GCST90027811.finngen_R12_E4_LACTONAS.pdf]

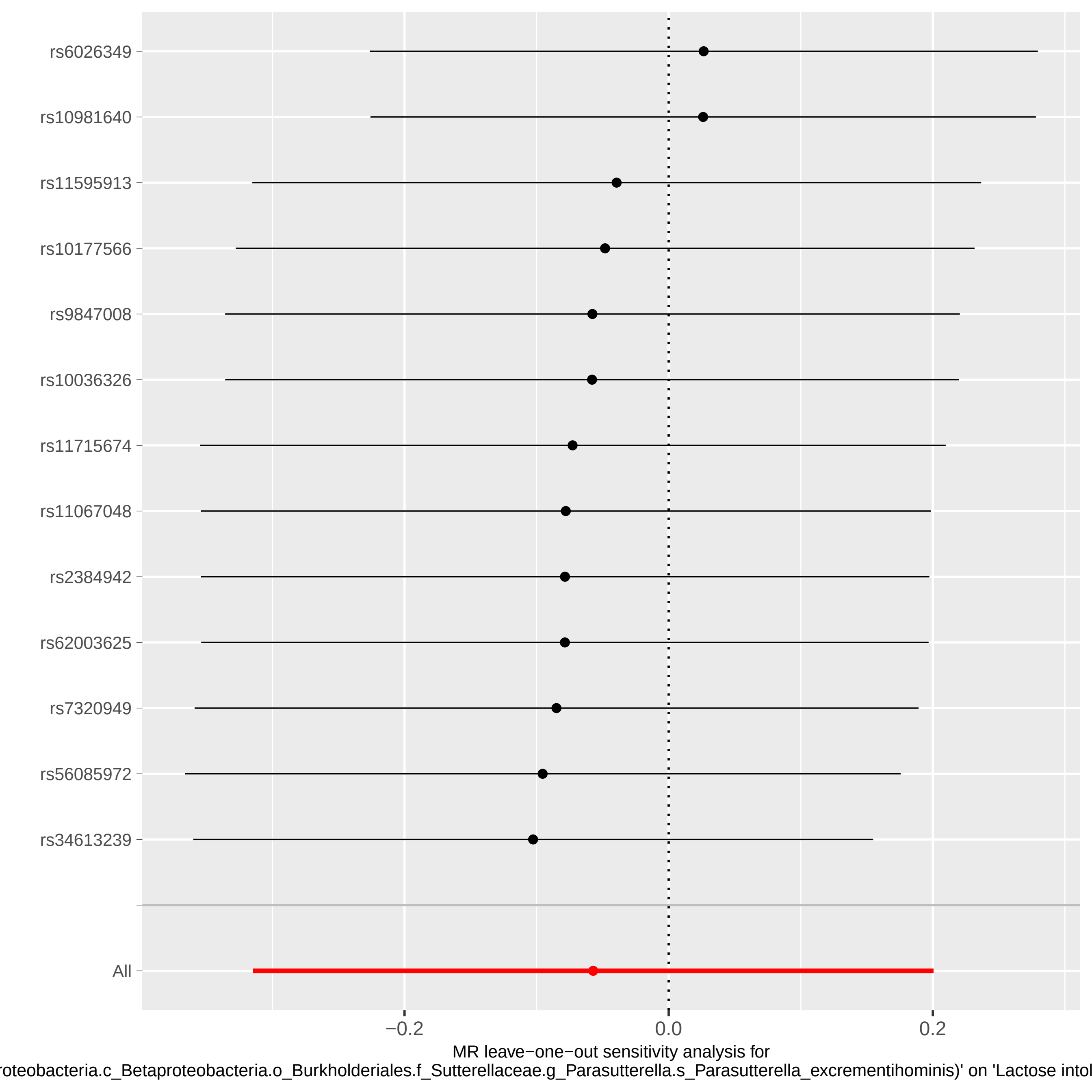
rs6026349
rs10981640
rs11595913
rs10177566
rs9847008
rs10036326
rs11715674
rs11067048
rs2384942
rs62003625
rs7320949
rs56085972
rs34613239
All
−0.2
0.0
0.2
MR leave−one−out sensitivity analysis for
roteobacteria.c_Betaproteobacteria.o_Burkholderiales.f_Sutterellaceae.g_Parasutterella.s_Parasutterella_excrementihominis)' on 'Lactose intol
